# Early Axonal Dysfunction of the Peripheral Nervous System Influences Disease Progression of ALS: Evidence From Clinical Neuroelectrophysiology

**DOI:** 10.3389/fneur.2021.574919

**Published:** 2021-02-11

**Authors:** Huiyan Yu, Lu Chen, Shuo Zhang, Jing He, Dongsheng Fan

**Affiliations:** ^1^Department of Neurology, Peking University Third Hospital, Beijing, China; ^2^Department of Neurology, National Center of Gerontology, Beijing Hospital, Beijing, China; ^3^Beijing Municipal Key Laboratory of Biomarker and Translational Research in Neurodegenerative Diseases, Beijing, China; ^4^Key Laboratory for Neuroscience, National Health Commission/Ministry of Education, Peking University, Beijing, China

**Keywords:** amyotrophic lateral sclerosis, compound muscle action potential, survival analysis, 12 months of symptom onset, peripheral nervous system

## Abstract

**Objective:** To assess the prognostic value of the decrement in compound muscle action potential amplitude within 12 months of symptom onset (CMAP-12 amplitude) for the survival of patients with amyotrophic lateral sclerosis (ALS).

**Methods:** Patients were stratified into 4 groups according to the decrement of the CMAP-12 amplitudes: normal (≥the lower limit of normal, LLN), mild (<LLN but ≥50% of LLN), moderate (<50% but ≥30% of LLN) and severe (<30% of LLN). All patients were followed up every 3 months. Survival was analyzed using the Kaplan-Meier method and Cox proportional hazards regression.

**Results:** A total of 149 patients were included in the analysis [90 males (60.4%); mean age at onset, 50.7 years]. The decrement of CMAP-12 amplitudes was normal in 24.2% of patients, mild in 22.1%, moderate in 15.4% and severe in 38.3%. Kaplan–Meier analysis showed there was a significant difference in the overall survival across the 4 groups (*p* < 0.05). Further pairwise comparisons identified significant differences in survival between the normal *vs*. the moderate group (*p* < 0.05) and the normal *vs*. the severe group (*p* < 0.01). There was a significant inverse correlation between the CMAP-12 amplitude and overall survival. Compared to that in the normal group, survival in the moderately and severely decreased groups was significantly shorter (HR 3.394, 95% CI 1.292–8.917, *p* = 0.013; and HR 4.732, 95% CI 2.032–11.017; *p* = 0.000, respectively).

**Conclusions:** Our results suggest that CMAP-12 amplitude could be a prognostic indicator of disease progression in ALS. More importantly, our findings provide clinical evidence for the viewpoint that early axonal dysfunction of the peripheral nervous system accelerates disease progression of ALS.

## Introduction

Amyotrophic lateral sclerosis (ALS) is a progressive neurodegenerative disease characterized by the degeneration of both upper and lower motor neurons. The prognosis and survival of ALS can vary between patients, as more than 60% of the patients die within 3 years of onset, while approximately 10% of the patients have a survival period of more than 8 years (1–4).

Although the fundamental mechanisms underlying ALS are not well-understood, current knowledge suggests that the main processes include impaired protein homeostasis, aberrant RNA metabolism, mitochondrial dysfunction, oxidative stress, glutamate excitotoxicity, nuclear export, impaired DNA repair, dysregulated vesicle transporters, glial dysfunction, and neuroinflammation. The increased excitability of motor nerves and axonal dysfunction are also considered to be pathophysiological mechanisms of ALS (2, 5–8). It has been shown that in the early stage of the disease, synaptic integrity and the stability of the distal cytoskeleton from the neuromuscular junction (NMJ) are destroyed, triggering a cascade of reactions toward the cell body (“dying-back” hypothesis). Furthermore, peripheral motor axons are involved in the pathogenesis and cascade of the development of ALS (9). It was proven that the altered axonal excitability potentially contributes to motor neuron death in ALS. It means that the axonal dysfunction rather than motor neuron loss in the anterior horn of the spinal cord is the cause of disease acceleration and that more axonal dysfunction seems predictive of a more aggressive phenotype (10–12).

The amplitude of compound muscle action potential (CMAP) is widely used in clinical practice as an important electrophysiological index to detect motor axonal damage (13). It was found that the decrease in the CMAP amplitude of the motor nerve in the early stage was significantly related to motor axonal hyperexcitability due to increased persistent sodium currents and reduced potassium currents in motor axons (5, 6, 14). To explore the relationship between motor axonal dysfunction in the early stage and in disease progression in patients with ALS, we studied the CMAP amplitude that was confined within 12 months of symptom onset (“CMAP-12 amplitude”) in these patients. We analyzed the relationship between the decrease in CMAP-12 amplitude and survival of patients with ALS. We hope to obtain clinical evidence that supports the view that the integrity of the peripheral nervous system (PNS) plays a protective role in the pathogenesis and progression of ALS in animal experiments (11, 12).

## Methods

### Participants

This study was a clinic-based prospective cohort study approved by the institutional ethics committee of the Peking University Third Hospital (IRB 00006761). Written informed consent was obtained from each patient.

Patients were recruited from January 2010 to December 2013, and each patient was given a follow-up evaluation by outpatient consultation or telephone every 3 months. For all cases, baseline demographic information and clinical data were collected during the patient's first visit and follow-up evaluations. Survival and tracheotomy were predefined as primary outcome measures.

Patients were diagnosed and classified according to the Airlie House diagnostic criteria (15). Those with pure lower motor syndromes were classified into an additional category of suspected ALS because they could not be classified using the established criteria (16). All patients were interviewed and examined by board-certified neurologists from the study group who had experience with ALS. According to the site of onset and clinical features, patients were categorized with limb-onset ALS, bulbar-onset ALS, flail arm syndrome (FAS)-type ALS (17), progressive muscle atrophy (PMA), or suspected primary lateral sclerosis (PLS) (those fulfilling all the diagnostic criteria of PLS in addition to the course of the disease were defined) (18). Since the etiology and prognosis of familial or juvenile ALS can be quite different from sporadic ALS, patients with familial and juvenile ALS were excluded from the analysis.

### CMAP Examination and Grouping

Clinical neurophysiologic examinations were carried out by using a Keypoint four-channel electromyography evoked potentiometer (Medtronic, USA). The motor nerve conduction of the median and ulnar nerves in the upper extremities as well as the peroneal and tibial nerves in the lower extremities were examined bilaterally by routine methods. The amplitude of the CMAP and nerve conduction velocity were recorded. All patients' sensory nerve conductions were also examined. The embedded pressure syndrome should be excluded. The examinations of all patients were strictly confined to be performed within 12 months of symptom onset including those performed within 6 months. For patients who underwent electrophysiological tests after 12 months, those with a normal amplitude were included in the normal group (since the amplitude remained normal within 12 months), while the other patients were not included in the study since it could not be clearly established whether the amplitude decline occurred within 12 months.

The CMAP-12 was recorded, respectively, from the abductor pollicis brevis, the abductor digiti minimi, the extensor digitorum brevis, or the flexor halluces brevis after median, ulnar, peroneal, or tibial nerve stimulation. The amplitudes of the CMAP-12 were stratified into four groups according to the nerve with the most obvious change: normal (≥ the lower limit of normal, LLN), mild decrease (<LLN but ≥50% of LLN), moderate decrease (<50% but ≥30% of LLN), and severe decrease (<30% of LLN). The ALS Functional Rating Score (ALS-FRS) and ALS Functional Rating Score Revised (ALS-FRS-R) were performed at the same time.

### Statistical Analysis

The data were collected to establish a database and SPSS 22.0 was used for data analysis (SPSS, Chicago, Illinois, USA). Continuous clinical and demographic variables that were normally distributed were compared using parametric tests (one-way analysis of variance, ANOVA), and categorical variables were analyzed using chi-square tests. The censoring date for the survival data was December 2017. Survival curves were estimated using Kaplan-Meier analysis, and covariates were compared using the log-rank test and Cox proportional hazards regression model.

## Results

A total of 149 patients with ALS were studied: including 90 males (60.4%) and 59 females (39.6%), with a male to female ratio of 1.5:1. The oldest onset age was 83 years old, and the youngest was 22 years old, with a mean age at symptom onset of 50.7 years.

Of 149 patients, 77 cases (51.7%) reached the end point, and 72 cases (48.3%) were censored, including lost to follow-up or living. The average survival time was 67.28 ± 5.33 months, and the median survival time was 56 ± 5.57 months. Of 36 cases (24.2%) with a normal CMAP-12 amplitude, 9 reached the end point, 27 cases were censored, and the median survival time was 111 ± 24.71 months. Of 33 cases (22.1%) with a mild CMAP-12 amplitude decrease, 13 cases reached the end point, 20 cases were censored, and the median survival time could not be calculated since fewer patients died; thus, the average survival time was 63.14 ± 5.3 months. Of 23 cases (15.4%) with a moderate CMAP-12 amplitude reduction, 13 cases reached the end point, 10 cases were censored, and the median survival time was 55 ± 14.48 months. Of 57 cases (38.3%) with a severe CMAP-12 amplitude reduction, 42 reached the end point, 15 cases were censored, and the median survival time was 40 ± 4.96 months.

There were 16 patients (10.7%) with bulbar onset, 85 patients (57.1%) with upper-limb onset and 48 patients (32.2%) with lower-limb onset. Regarding the phenotypes, 120 patients (80.5%) had the typical limb-onset of ALS, 14 patients (9.4%) had the bulbar-onset of ALS, 8 patients (5.4%) had FAS-type ALS, 4 patients (2.7%) had PMA, and 3 patients (2%) had suspected PLS. Diagnostic grades included 37 patients (24.8%) as definite, 39 patients (26.2%) as probable, 43 patents (28.9%) as laboratory-supported probable, 25 patients (16.8%) as possible, and 5 patients (3.3%) as suspected ALS.

Demographics and clinical characteristics of patients with ALS who had different severities of decrements of CMAP amplitude within 12 months of symptom onset, i.e., CMAP-12 amplitudes, are shown in Table 1. There was an overall difference in the onset sites among the four groups (*p* < 0.01), with more bulbar-onset patients in the normal group and more limb-onset patients in the moderately or severely decreased CMAP-12 amplitude groups. The scores of the ALS-FRS and ALS-FRS-R were lower in the severely decreased CMAP-12 amplitude group than in the normal and mildly decreased groups (*p* < 0.01), but there were no significant differences between the other groups (Table 1). There were no significant differences in the sex ratio, onset age, phenotype of disease, and diagnostic grade among the four groups.

**Table 1:** Demographics and clinical characteristics of patients with ALS with different severities of decrements of CMAP-12 amplitude.

	**Normal**	**Mild**	**Moderate**	**Severe**	**Total**	***p*-value**
Total *n* (%)	36 (24.2)	33 (22.1)	23 (15.4)	57 (38.3)	149	
Age at onset	51 ± 10.3	51.4 ± 13.0	50.5 ± 9.9	50.3 ± 10.0		0.964
Sex ratio (M:F)	1.4:1	1.4:1	0.9:1	2.2:1	1.5:1	0.367
Onset site, *n* (%)						<0.0001
Bulbar	9 (25.0)	6 (18.2)	1 (4.3)	0	16 (10.7)	
Upper limbs	14 (38.9)	23 (69.7)	16 (69.6)	32 (56.1)	85 (57.1)	
Lower limbs	13 (36.1)	4 (12.1)	6 (26.1)	25 (43.9)	48 (32.2)	
Phenotype, *n* (%)						0.066
Limb-onset ALS	24 (66.7)	25 (75.8)	19 (82.6)	52 (91.2)	120 (80.5)	
Bulbar-onset ALS	8 (22.2)	5 (15.1)	1 (4.3)	0	14 (9.4)	
FAS-type ALS	1 (2.8)	2 (6.1)	2 (8.7)	3 (5.3)	8 (5.4)	
PMA	2 (5.5)	0	1 (4.3)	1 (1.8)	4 (2.7)	
Suspected PLS	1 (2.8)	1 (3.0)	0	1 (1.8)	3 (2.0)	
Airlie house category (%)						0.399
Clinically definite	7 (19.4)	7 (21.2)	7 (30.4)	16 (28.1)	37 (24.8)	
Clinically probable	6 (16.7)	7 (21.2)	9 (39.1)	17 (29.8)	39 (26.2)	
Laboratory-supported probable	15 (41.7)	9 (23.3)	3 (13.1)	16 (28.1)	43 (28.9)	
Clinically possible	6 (16.7)	9 (27.3)	3 (13.1)	7 (12.3)	25 (16.8)	
Suspected	2 (5.5)	1 (3.0)	1 (4.3)	1 (1.7)	5 (3.3)	
FRS score	34.6 ± 4.6	35.3 ± 3.6	33.6 ± 4.0	31.7 ± 5.1		0.001
FRS-R score	42.3 ± 5.0	43.2 ± 3.7	41.7 ± 4.0	39.1 ± 5.4		0.001

**Figure 1:**
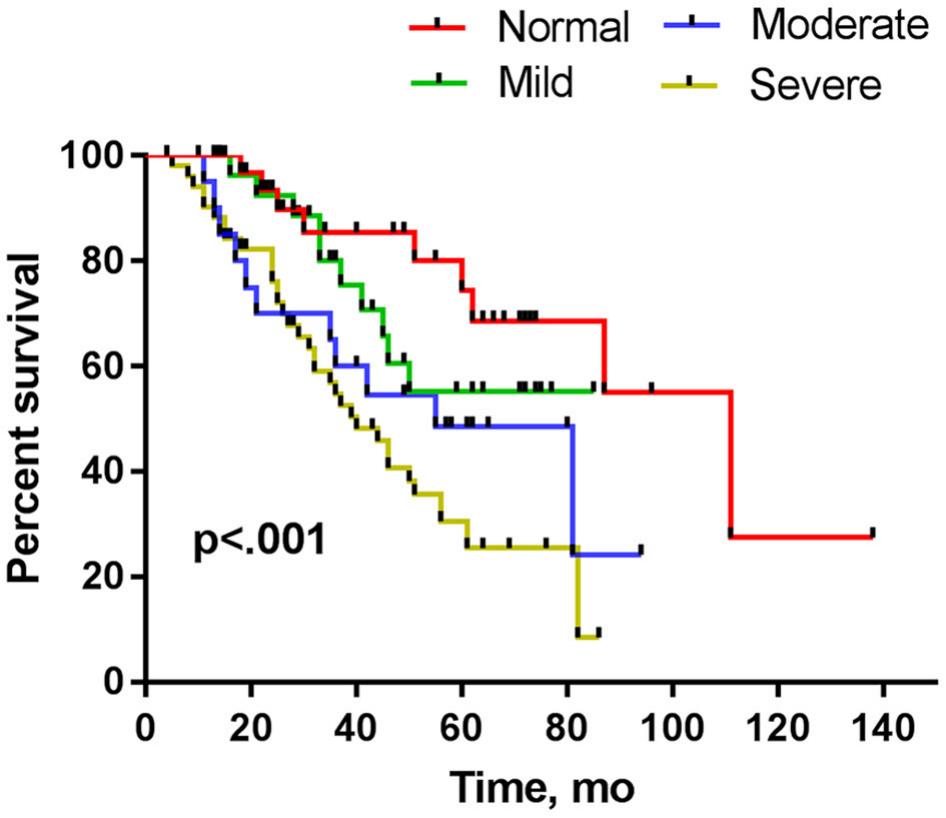
Kaplan–Meier plots of surviving patients stratified by decrements in CMAP amplitude assessed within 12 months from disease onset.

In Kaplan–Meier analysis, sex, onset site, phenotype of disease, and diagnostic grade had no significant impact on survival (*p* > 0.05), but the CMAP-12 amplitude had a significant effect on survival. There was a significant difference in the overall survival in the four groups (*p* < 0.05; Table 2). Further pairwise comparisons among different categories of CMAP-12 amplitude revealed a statistically significant difference in survival between the normal group and the moderately decreased group (*p* < 0.05) and between the normal and severely decreased groups (*p* < 0.01). There was also a significant difference in survival between those with mildly decreased CMAP-12 amplitude and those with severely decreased CMAP-12 amplitude (*p* < 0.05; Table 3). There were no other differences among the groups. The Kaplan-Meier survival plots are presented in Figure 1.

**Table 2:** Kaplan–Meier survival analysis for sex, onset site, phenotype, diagnostic grade, and CMAP.

	**Category**	**Median survival (month)**	**χ^2^**	***p*-value**
Sex	Male	55	3.73	0.053
	Female	81		
Onset site	Bulbar	87	0.083	0.893
	Upper limb	56		
	Lower limb	61		
Phenotype	Limb onset	56	0.599	0.439
	Bulbar onset	87		
	FAS-type ALS	37		
	PMA	46		
	suspected PLS			
Airlie house category	Definite	81	0.001	0.978
	Probable	55		
	Lab-supported probable	60		
	Possible	50		
	Suspected	46		
CMAP	Normal	111	17.428	0.000^a^
	Mild	N		
	Moderate	55		
	Severe	40		

a *p ≤ 0.01*.

**Table 3:** Log-rank (Mantel-Cox) pairwise comparisons in different categories of decreased CMAP amplitude.

	**Normal**		**Mild**		**Moderate**		**Severe**	
	**χ^2^**	***p***	**χ^2^**	***p***	**χ^2^**	***p***	**χ^2^**	***p***
Normal			1.183	0.277	4.790	0.029^a^	15.109	0.000^b^
Mild	1.183	0.277			0.967	0.325	6.465	0.011^a^
Moderate	4.790	0.029^a^	0.967	0.325			1.364	0.243
Severe	15.109	0.000^b^	6.465	0.011^a^	1.364	0.243		

a *p ≤ 0.05*,

b *p ≤ 0.01*.

**Figure 2:**
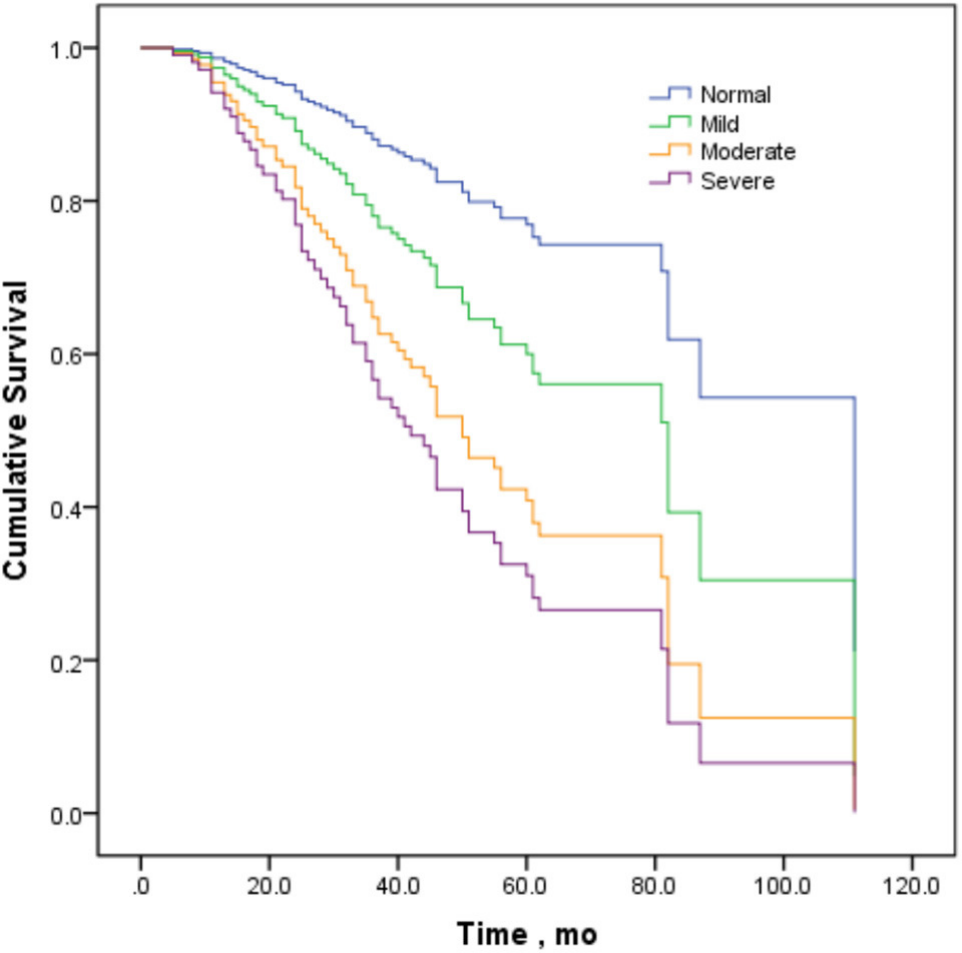
Survival curves stratified by decrements in CMAP amplitude in the Cox proportional hazard regression model.

The variables of sex, onset site, diagnostic grade, CMAP-12 amplitude, and ALSFRS-R were included in the Cox regression model except that phenotype was not included due to fewer cases in some categories. The Cox proportional hazard regression analysis confirmed a significant inverse correlation between CMAP-12 amplitude and overall survival (*p* < 0.05), adjusting for the confounding effects of sex, onset site, and diagnostic grade. The change in CMAP-12 amplitude was correlated with prognosis, and compared to the normal group, the survival of the moderately decreased group was significantly shorter (p < 0.05), and the survival in the severely decreased group was the shortest (*p* < 0.01; Table 4, Figure 2). These data indicated that a decrease in CMAP-12 amplitude was an independent risk factor for fast progression. The Cox regression model also showed a poorer prognosis for patients with lower ALSFRS-R (*p* < 0.05; Table 4).

**Table 4:** Cox proportional hazard regression analysis.

**Variable**	**HR**	**95%CI**		***P*-value**
		**Lower**	**Upper**	
**Sex**				
Male	1			
Female	0.659	0.379	1.148	0.141
Age at onset	1.014	0.988	1.040	0.288
**Onset site**				
Bulbar	1			
Upper-limb	0.814	0.316	2.094	0.669
Lower-limb	0.500	0.178	1.401	0.187
**Airlie house category**				
Clinically definite	1			.
Clinically probable	1.411	0.679	2.932	0.356
Laboratory-supported probable	1.415	0.653	3.065	0.379
Clinically possible	2.110	0.855	5.205	0.105
Suspected	1.334	0.286	6.226	0.714
ALSFRS-R	0.929	0.878	0.984	0.012^a^
**CMAP-12**				
Normal	1			
Mild	1.924	0.715	5.178	0.195
Moderate	3.394	1.292	8.917	0.013^a^
Severe	4.732	2.032	11.017	0.000^b^

a *p ≤ 0.05*,

b *p ≤ 0.01; HR, Hazard Ratio; CI, Confidence Interval*.

The patients with moderately and severely decreased CMAP-12 amplitude were further divided into two subgroups: recorded within 6 months (31 cases) and within 12 months (49 cases). The Kaplan-Meier survival analysis showed that the median survival time of the 6-month group was 27 months, and that of the 12-month group was 55 months. The survival time of the 6-month group was significantly shorter than that of the 12-month group (*p* = 0.015).

## Discussion

Our study found that CMAP-12 amplitudes in patients with ALS had remarkable divergences. Eighty patients (53.7%) had CMAP-12 amplitudes decreased by more than 50%, suggesting that approximately half of the patients had obvious motor axon dysfunction in the early stage of the disease. From the ALS-FRS and ALS-FRS-R scores, we can see that the severity of the disease in the moderately and severely decreased, but not the mildly decreased, groups was significantly worse than that of the normal group. This confirms that motor axonal dysfunction could occur in the relatively early stage of ALS and that a greater decrease in CMAP amplitude is associated with more deterioration in clinical function.

The univariate survival analysis showed that the reduction in the CMAP-12 amplitude was significantly correlated with survival. A further decrease in the CMAP-12 amplitude was associated with a further shorter survival. Multivariate analysis, after adjusting for the influence of multiple confounding factors, further found that the CMAP-12 amplitude remained closely related to survival. The survival time of patients with moderately and severely decreased CMAP-12 amplitude was significantly shorter than that of the normal group, with the severely decreased CMAP-12 associated with the shortest survival. The results indicated that the decrement in CMAP amplitude within 12 months of onset can be used as an independent prognostic factor. Furthermore, our subgroup analysis showed that the survival period was shorter in the patients with moderately and severely decreased CMAP amplitudes within 6 months than in patients with similar decreases within 12 months, suggesting that the earlier the amplitude of CMAP decreased, the worse the prognosis.

There have been studies evaluating the CMAP amplitude, neurophysiological index (NI), motor unit number estimation (MUNE), and motor unit number index (MUNIX) as electrophysiological markers for the prognosis of the disease (12, 19–26). Imai et al. reported that CMAP amplitude had the strongest influence on prognosis outside the onset site. The higher the median nerve CMAP amplitude is, the better the prognosis in all age groups, suggesting that the CMAP amplitude is a valuable indicator of prognosis of ALS (27). Our present study differs from previous studies in defining the assessment time for CMAP changes in patients to within 12 months of onset, based on the fact that the median survival time of patients with ALS is generally 2 to 4 years, and 12 months may be regarded as a relatively early stage of the disease (28–30). As the disease progresses, all patients will undergo a decrease of CMAP amplitude to varying degrees. Especially in the late stage of the disease, almost all patients have an obvious reduction of CMAP amplitude due to substantial motor neuron loss. From our previous study and the data in this study, we found the reduction of the CMAP amplitude was diverse within 12 months of onset. We believe that it is more meaningful to study the changes of CMAP amplitude in patients with ALS in the early stages of the disease than during the whole course of the disease. We speculate that the mechanism of the decline in CMAP-12 amplitude may consist of two components: (1) hyperexcitability of peripheral motor axons, and (2) motor axonal damage associated with earlier motor neuronal death (6–8). In addition to considering the early stage (within 12 months of onset), we used a much more routine electrophysiological method and selected the most obvious changeable nerve, illustrating the highlights of our study.

Fischer et al. found that denervated synapses of IIb/x muscle fibers in ALS SOD1 mice (47 days old) and axonal loss of the ventral peripheral nerve (80 days old) occurred before neuronal cell body loss (100 days old) (31). It was found that proximal axonopathy in ALS is associated with the loss of neurofilament (NF) protein in the terminal neuromuscular junction as well, and the progressive loss of NFs may evolve from distal to proximal (32). In recent years, a dying-back hypothesis has attracted much attention (10, 31, 33, 34). According to this hypothesis, motor nerves and nerve endings exhibit pathophysiological changes before degeneration of motor neurons and clinical symptom onset in some patients. In particular, the hypothesis suggests that ALS could be a distal axonopathy and that NMJ function may change first and then the pathophysiological changes progress proximal to the cell body (34). Evidence of dying-back was found in an autopsy of a patient with ALS with denervation and innervation of the muscles, while no pathological changes occurred in the motor neurons themselves (31). Recent studies have found that stimulated Raman scattering (SRS) microimaging can sensitively detect peripheral nerve degeneration in ALS mice and pathological specimens from patients with ALS. It was also found that clear degeneration of the peripheral nerve appeared at the same time as denervated muscle in an early clinical mouse model and that these changes occurred earlier than hypofunction of the motor nerve (35).

Nardo et al. found that, compared with C57SOD1^G93A^ mice with slow disease progression, 129SvSOD1^G93A^ mice with rapid disease progression had significant peripheral axonal loss during the onset of the disease, which suggested that PNS damage rather than motor neuron loss itself was related to the rapid progression of the disease (11, 12). Grouping CMAP amplitudes as determined within 12 months and comparing the differences among groups could help to sort out patients with different patterns of disease progression and to study the underlying mechanism of this progression and even apply drug trials in the future. Our study supported that peripheral motor axonal dysfunction can occur in the early stage of ALS and that the degree of the axonal injury was related to disease progression and survival. Maintaining peripheral nerve integrity is essential to slow down the progression of the disease (12), which may provide a clue for future drug development.

In this study, the percentage of bulbar-onset patients was lower; however, this result is consistent with our previous study (4). In 16 patients with bulbar onset, there were 14 patients of bulbar-onset type ALS, 1 patient of PMA and 1 patient of PLS. It showed a higher proportion of bulbar-onset patients in the normal CMAP-12 amplitude group than in the other groups. The median survival time of 14 patients of bubar-onset ALS was 87 months, which seems to contradict the common belief that this type of patient usually has a poorer prognosis than those with limb-onset ALS. However, it is noteworthy that in 14 patients with bulbar-onset ALS, there are five patients (35.7%) that conform to the isolated bulbar phenotype of ALS (IBALS) (36, 37). A high proportion of patients with IBALS (5/14) may partly explain why the median survival of the patients with bulbar-onset ALS was not different than those with limb-onset ALS in this study (Table 2). The other reason may be data bias due to the small sample size. On the other hand, although Kanai et al. showed the motor axonal excitability properties were strong and independent predictors for shorter survival in patients with ALS, they did not find differences of the prognosis by multivariate analysis between the bulbar-onset and non-bulbar-onset patients in their study (7). The electromyography damage of patients with IBALS does not meet the Awaji criteria for the diagnosis of the “clinical definite” or “clinical probable” grade of patients with ALS. There was no significant difference in CMAP amplitude of IBALS compared with the limb-onset group even though there were 4 IBALS with a mild CMAP-12 decline. The results showed that the CMAP decrease of patients with IBALS was not obvious in the early stage of the disease, but we note that although IBALS clinical symptoms are confined to the bulbus for 6 months, the electrophysiological examination in the early stage may reveal abnormalities beyond the bulbus, which may precede the clinical symptoms. Because of the small sample size of bulbar-onset patients in our study, the differences of CMAP-12 amplitude decline or survival between patients with IBALS and those with non-IBALS cannot be compared, and thus these facets of IBALS deserve further study in the future.

There were 8 patients with FAS ALS in this study whose median survival (36 months) was shorter than other clinical types. It seems to be inconsistent with the generally considered milder course of disease in FAS. FAS diagnosis usually follows the “12 month” standard proposed by Wijesekera (16), defining FAS as having symptoms confined to both upper limbs at least 12 months after onset, with no bulbar and lower-limb symptoms. However, there are other studies that define FAS time as 18 months after onset or at least 12 months after the first visit. Whether the definition of at least 12 months after onset is reasonable has not yet been determined. Moreover, in our ALS database, patients with ALS who limit their symptoms to the upper limb within 12 months of onset are defined as FAS-type ALS (FAS type). A recent study published by our team (17), which compared the survival time of patients who met the diagnostic criteria of Wijesekera with patients with FAS-type ALS (that is, the onset of the disease was limited to the upper limb for 12 months). The median survival time of patients with FAS-type ALS (~35 months) was shorter than that of patients with FAS (~89 months). From that study, we found that as a long-lived, milder specific clinical phenotype, it is reasonable to define FAS as at least 12 months after onset. In this study, we included patients with ALS within 12 months of onset, which can be only classified as FAS-type ALS. We found that the median survival of the patients with FAS-type ALS was shorter (36 months) (Table 2) This result was consistent with our previous study (17). Some FAS-types may actually be a subtype of limb-onset ALS, but because the symptoms are limited to the upper limb for a long time, they are somewhat different from the classical limb-onset type, so they are listed separately. FAS is a very special phenotype, and it can have early CMAP reduction, but the prognosis is good. These findings and characteristics show that there are more underlying complicated mechanisms that are also interesting and worth exploring.

Biopsy for patients with ALS is not ethical since it can damage motor nerves. It is difficult to distinguish whether CMAP amplitude decrements derive primarily from motor neuron loss or axonal degeneration due only to electrophysiology. However, based on the theory of neuromuscular junction origin and dying-back, there may be a process of degeneration of motor neurons from distal to proximal; that is, axonal degeneration is earlier than the loss of motor neurons in the early stages of some patients with ALS. Unlike in the later stages, we should not classify all CMAP decline as loss of motor neurons. We hope that this study will raise this issue, attract more attention, and promote additional research on the mechanism of CMAP decline and axonal protection in the early stage of ALS.

This study has several limitations. First, the sample size of patients was limited, especially patients in the moderately decreased CMAP-12 amplitude group. Second, important data on the use of noninvasive ventilation, riluzole or gastrostomy and pulmonary function were absent in this study. Third, we studied only the CMAP amplitude of motor nerves. Recently, Miyaji et al. showed that the attenuation rate of repetitive nerve stimulation (RNS) was negatively correlated with the CMAP amplitude of the first wave. The higher the RNS attenuation rate was, the lower the CMAP amplitude, which likely indicates that there was a correlation between terminal axonal dysfunction and NMJ injury (38). In the future, we might need to combine the measurements of the CMAP-12 amplitude with NMJ changes in patients with ALS to better understand the origin and mechanisms of ALS.

In summary, our data support the previous animal studies from a clinical electrophysiological point of view by showing that the severity of the decrements of CMAP amplitude in the early stage of ALS is significantly correlated with the severity of the disease. The decrement in CMAP amplitude within 12 months of symptom onset (CMAP-12 amplitude) could be an electrophysiological marker to predict disease progression and survival.

## Data Availability Statement

The raw data supporting the conclusions of this article will be made available by the authors, without undue reservation.

## Ethics Statement

The studies involving human participants were reviewed and approved by the institutional ethics committee of the Peking University Third Hospital (IRB 00006761). The patients/participants provided their written informed consent to participate in this study.

## Author Contributions

DF and HY conceived and designed the study. LC, SZ, and JH performed the experiments. HY wrote the manuscript. DF reviewed and edited the manuscript. All authors read and approved the manuscript.

## Conflict of Interest

The authors declare that the research was conducted in the absence of any commercial or financial relationships that could be construed as a potential conflict of interest.
